# Effects of a thermoelectric element tourniquet on venipuncture pain and stress relief in Korea: a randomized controlled trial

**DOI:** 10.7586/jkbns.25.018

**Published:** 2025-05-23

**Authors:** Tae Jung Lee, Jihoo Her, Myung-Haeng Hur

**Affiliations:** 1 Uijeongbu Eulji University Hospital, Uijeongbu, Korea; 2 College of Nursing, Gimcheon University, Gimcheon, Korea; 3 College of Nursing, Eulji University, Uijeongbu, Korea

**Keywords:** Cryotherapy, Hyperthermia, induced, Injections, intravenous, Pain, Tourniquets

## Abstract

**Purpose:**

This study developed a thermoelectric element (TEE) tourniquet integrating a tourniquet with a temperature control device capable of delivering heat or cold therapy. A randomized controlled trial was conducted to evaluate the effects of the TEE tourniquet on pain, stress, and satisfaction during venipuncture.

**Methods:**

In total, 118 hospitalized adults were randomly assigned to heat therapy (40~45°C), cold therapy (0~10°C), thermal grill illusion therapy (alternating heat and cold), or the control group. The TEE tourniquet was applied 10 cm above the puncture site. A temperature intervention began 5 seconds before cannulation and was maintained during the procedure, typically lasting 10 to 30 seconds. The control group received the TEE tourniquet without temperature activation. Outcomes included perceived pain and stress (numerical rating scale), observed pain (Wong-Baker FACES), SpO_2_, stress index, and participant satisfaction.

**Results:**

Significant differences were found among groups in perceived pain (F = 4.82, *p* = .003), observed pain (F = 5.50, *p* = .001), and perceived stress (F = 4.72, *p* = .004). The heat therapy group reported significantly lower pain and stress than the control group. No significant differences were found in SpO_2_, the stress index, or satisfaction.

**Conclusion:**

Heat therapy via the TEE tourniquet significantly reduced venipuncture-related pain and stress. Given its short application time and usability, this device may serve as a clinically useful nursing intervention to improve comfort during invasive procedures.

## INTRODUCTION

Venous catheterization is one of the most frequent invasive procedures performed by nurses in hospitals [1], and the peripheral venous route for fluid infusion is used in up to 80% of hospitalized patients [2]. However, pain relief tends to be overlooked, as venipuncture pain is a transient part of treatment [3]. Despite this, many hospitalized patients fear needling, and discomfort due to venipuncture is one of the most common concerns in healthcare facilities [4]. In addition to pain, venipuncture often elicits psychological stress, particularly in individuals with heightened sensitivity to medical procedures. Stress can exacerbate pain perception through the activation of the hypothalamic–pituitary–adrenal axis and sympathetic nervous system, which can increase procedural discomfort and negatively affect patient cooperation [5] However, studies focusing on stress relief during venipuncture remain limited. Regardless of the cause, managing and minimizing patient pain is an essential nursing responsibility [6].

Most studies on reducing pain and stress during venipuncture have focused on pediatric patients [7-9]. These studies primarily investigated visual or auditory distractions, such as video games, comic books [10,11]; tactile stimulation with a vibrating ice pack [7,12]; and topical interventions, including anesthetic cream or vapocoolant spray [13,14]. In adult patients, studies have investigated the effects of heat or cold therapy, topical anesthetic cream, vapocoolant spray and tactile stimulation [15-20].

These interventions alleviate pain and stress through various mechanisms. Distraction techniques such as video games or comic books reduce perceived pain by diverting attention from the needle insertion, thereby modulating pain perception through cognitive engagement [10,11]. Tactile stimulation and vibration interfere with pain signal transmission via the gate control theory of pain [7,12]. Topical anesthetics block nerve conduction at the puncture site, reducing local sensitivity [13,14]. Similarly, cold application induces vasoconstriction and slows nerve conduction velocity, while heat increases blood flow and reduces muscle tension, both contributing to decreased discomfort [15-18].

Heat and cold therapies have been applied as cost-effective non-drug interventions that nurses can perform independently, without a doctor’s prescription. Heat therapy may include the use of a water bottle, clay pack, or electrical pad [21]. Cold therapy may include the application of a polyvinyl chloride bag with saline [17], a cold and vibration device [22], or an ice pocket [23]. However, considering the short time required for venipuncture (within 1 minute), these therapies may be inconvenient as they need to be prepared in advance, limiting their widespread use in clinical practice.

One method that enables more efficient application of thermal therapy is the recently developed thermoelectric element (TEE) technique, which uses a module that converts electrical energy into thermal energy. Another approach is the thermal grill illusion (TGI), which creates the sensation of pain through alternating heat and cold stimuli. Although the stimuli are harmless, they induce a sensory illusion that mimics real pain [24]. When TGI was applied to patients with chronic pain, pain and discomfort were significantly reduced [25], and its analgesic effects have also been confirmed in pharmacological studies [26].

Recently, a study examined the effect of a TEE band in venipuncture for blood sampling for the first time in South Korea [27]. In this study, cold, heat, and TGI therapies were applied to an area close to the venipuncture site. Despite the lack of pain relief, participants' satisfaction increased with cold therapy. However, the use of both a tourniquet and TEE band during venipuncture was shown to increase the inconvenience for nurses, as this required an additional procedure, and it was also pointed out that further studies should be conducted to determine the inconsistencies in the distance between the venipuncture site and treatment area among patients.

Thus, this study aimed to develop a TEE tourniquet with a temperature control function by combining a tourniquet with a temperature control device that can apply heat, cold, and TGI therapies. The effects of our TEE tourniquet on pain and stress were comparatively analyzed to evaluate its potential as an effective and practical nursing intervention for routine clinical use.

## METHODS

### 1. Study Design

**Figure 1.**
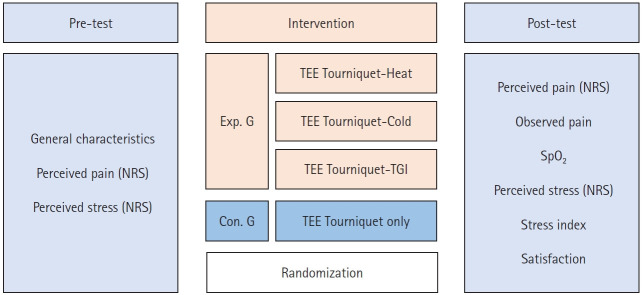
Study design. Exp. G = Experimental group; Con. G = Control group; TEE Tourniquet-Heat = Thermoelectric element tourniquet- heat group; TEE Tourniquet-Cold = Thermoelectric element tourniquet-cold group; TEE Tourniquet-TGI = Thermoelectric element tourniquet-thermal grill illusion group; TEE Tourniquet only = Thermoelectric element tourniquet group; NRS = Numeric rating scale; SpO_2_ = Saturation of percutaneous oxygen.

This study had a randomized controlled pre-test–post-test design to determine the effects of the TEE tourniquet on pain, stress, and satisfaction in the heat, cold, TGI, and control groups (use of the TEE tourniquet only, without heat, cold, or TGI therapy). The study design is illustrated in Figure 1.

### 2. Participants

Among the patients admitted to the general wards at Daejeon Eulji Medical Center, South Korea, those who received venipuncture with an 18-gauge angiocatheter (1.16 inch) for fluid infusion were recruited. Participants were recruited via convenience sampling based on voluntary responses to an internal hospital advertisement. The inclusion criteria were adults aged 20–70 years who had undergone venipuncture within the past 6 months, were able to communicate, and had no hypersensitivity to heat or cold therapy or any circulatory disease. The 6-month timeframe was selected to minimize recall bias while ensuring participants had a recent and relevant venipuncture experience. Patients who had received systemic analgesics, were undergoing other pain management treatments, or had a diagnosed mental disorder were excluded.

**Figure 2.**
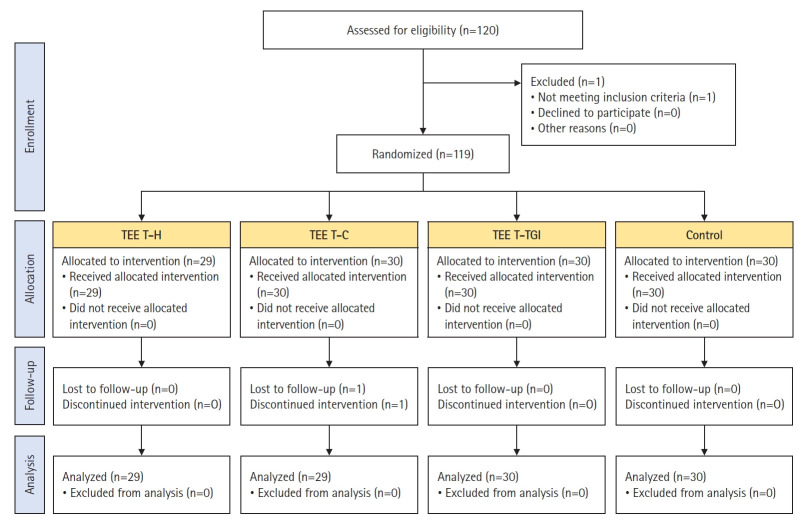
Flow diagram. TEE T-H = Thermoelectric element tourniquet- heat group; TEE T-C = Thermoelectric element tourniquet-cold group; TEE T-TGI = Thermoelectric element tourniquet-thermal grill illusion group; Control = Control group.

The sample size was estimated based on a previous study, using an F-test and ANOVA in G*Power 3.1.9.4. With an effect size of 0.32 [28], a significance level of 0.05, power of 0.80, and four groups, a sample size of 108 was required. Considering a 10% dropout rate, 120 participants were recruited. One participant was excluded due to not meeting the venipuncture experience criterion, and the remaining were randomized into the heat (n = 29), cold (n = 30), TGI (n = 30), and control (n = 30) groups. One participant in the cold group withdrew during the study, resulting in 29 participants each in the heat and cold groups, and 30 each in the TGI and control groups. The participant flow is illustrated in Figure 2.

#### 1) Participant bias

To prevent participant bias in this study, each participant was coded in the order of recruitment, and group assignment was conducted using the RAND function in Microsoft Excel, which generated random numbers to allocate participants to one of the four groups. In addition, the participants were not given any information on the group they belonged to, and Assistant 2, who rated the dependent variables, was blinded; however, complete blinding was a challenge due to the characteristics of the study.

#### 2) Training of research assistants

The two assistants in this study were trained by the principal investigator in two one-hour sessions, which covered the study purpose, rationale, and procedures related to the experimental interventions. Assistant 1 was recruited from among nurses with at least 15 years of clinical experience to prevent the occurrence of any issues related to the venipuncture technique; this nurse was the one who performed the TEE tourniquet intervention. Assistant 2, a nurse with ≥10 years of clinical experience, was the one who rated the dependent variables of saturation of percutaneous oxygen (SpO_2_), stress index, and pain relief during venipuncture.

### 3. Instruments and Measurements

#### 1) Pain

Pain was assessed using three methods to capture different dimensions of the pain experience: (1) perceived pain, based on the participant’s self-reported intensity; (2) observed pain, rated by a trained assessor; and (3) physiological response, measured via oxygen saturation (SpO_2_), which was included as a supplementary indicator given its feasibility for non-invasive monitoring during the procedure. Although both perceived and observed pain used a 0~10 scale, they reflect distinct aspects of pain and were included to ensure a multidimensional assessment [29,30].

***Perceived pain*:** The numeric rating scale (NRS) was used to measure the perceived pain levels. The NRS for perceived pain was set on a horizontal line, starting from 0 (no pain at all) and ending with 10 (severe pain), with equal intervals between the scores. The participants were instructed to mark the score that represented their pain level, with higher scores indicating higher pain levels.

***Observed pain*:** The Wong-Baker FACES Pain Rating Scale was used to measure the level of pain observed during venipuncture for each patient. In addition, a research assistant rated the level of pain felt by the participant on the NRS, as described above. The assistant marked the score for observed pain, with higher scores indicating higher levels of pain.

***SpO_2_ (physiological response)*:** A pulse oximeter (32MX, Nellcor, Covidien, USA) was applied to the tip of the index finger of the contralateral arm to the one being punctured to measure peripheral oxygen saturation as a physiological indicator of pain [31].

#### 2) Stress

***Perceived stress*:** The NRS was used to measure perceived stress levels. The NRS for perceived stress was set on a horizontal line from 0 (no stress at all) to 10 (severe stress), with equal intervals between scores. The participants were guided to mark the score to represent the stress level they felt, with higher scores indicating higher stress levels.

***Stress index*:** Canopy 9 RSA (IEMBIO, Korea), a device that measures autonomic nervous system activity, was used to measure the stress index. The sensor of the Canopy 9 RSA was attached to the tip of the index finger on the arm contralateral to the one being punctured to measure. The stress level ranged between 1 and 10, with higher numbers indicating higher stress levels.

#### 3) TEE tourniquet satisfaction

After the experimental intervention, the participants’ satisfaction with the TEE tourniquet was measured using a questionnaire that was used in a previous study (Cronbach’s α = .87) [27]. The questionnaire consisted of ten items, including the level of pain reduction, satisfaction, and intention to reuse. Each item was rated on a 5-point Likert scale, using scores ranging from 1 (strongly disagree) to 5 (strongly agree), with higher scores indicating higher levels of satisfaction. The reliability of this tool in this study was Cronbach’s α = .94.

### 4. Experimental interventions

In this study, venipuncture was performed on the participants using a TEE tourniquet that applied heat, cold, or TGI therapy.

#### 1) TEE tourniquet preparation

The TEE tourniquet used in this study included a flexible thermoelectric device that could generate heat and cold through an exothermic or endothermic mechanism according to the direction of the current through a grill-type plate (5.5 cm × 3.5 cm). It contained an elastic material, so it could be applied to the upper or lower part of the arm. The plate temperature was set according to that recommended in previous studies that used an ice pocket, hand warmer, or electric heating pad for fomentation as intervention tools to reduce pain at the venipuncture site [7-10,27]. For cold therapy, prior studies recommend maintaining temperatures between 0~10°C, typically applied for a short duration without adverse effects [32]. For heat therapy, effective pain relief has been demonstrated at 40~45°C under similar conditions [27,33]. Based on these findings, the TEE tourniquet was configured in accordance with the recommended temperature ranges to ensure effective pain relief while minimizing the risk of skin burns or tissue damage.

**Figure 3.**
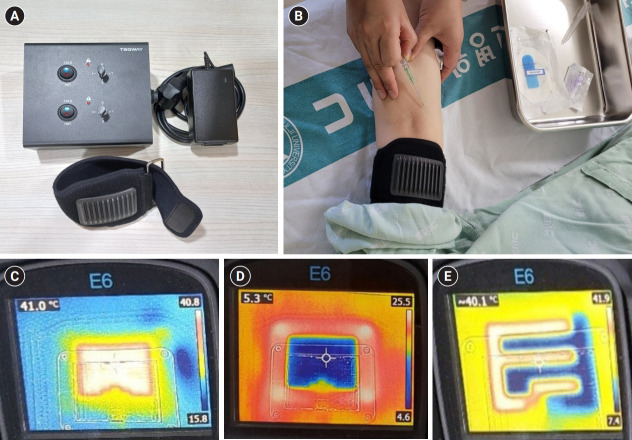
Applying the thermoelectric element (TEE) tourniquet intervention. (A) TEE tourniquet and controller; (B) Applying the TEE tourniquet intervention during venipuncture; (C) Plate temperature when applying TEE tourniquet heat therapy (40~45°C); (D) Plate temperature when applying TEE tourniquet cold therapy (0~10°C); (E) Plate temperature when applying TEE tourniquet thermal grill illusion therapy (40~45°C, 0~10°C).

A clinical nurse with 15 years of clinical experience and a high level of venipuncture practice, a professor in the nursing department, a chief technology officer majoring in material engineering, and the developer of the flexible thermoelectric device participated in the development of the TEE tourniquet. Several discussions were held on the shape of the tourniquet, the size of the plate responsible for the flexible thermoelectric mechanism, and temperature range. A thermal imaging camera (FLIR, E6390, Sweden) was used to check the temperature achieved by the plate in the TEE tourniquet for each use (Figure 3).

#### 2) TEE tourniquet application

Participants who met inclusion criteria and consented to participate completed a pre-test questionnaire assessing general characteristics and baseline levels of perceived pain and stress (NRS) related to prior venipuncture experience. The stress index using Canopy 9 RSA was measured only after venipuncture, as it is designed to capture immediate autonomic responses during the procedure.

Venipuncture was performed in a general ward bed with a room temperature maintained between 22~25°C. To reduce circadian influence, all procedures were conducted between 9:00 AM and 3:00 PM. Efforts were made to minimize distractions from environmental variables such as noise, lighting, and staff movement.

Assistant 1 applied the TEE tourniquet within 10 cm of the puncture site and activated the designated thermal setting. The site was disinfected, and venipuncture was carried out using an 18-gauge angiocatheter. Thermal stimulation began 5 seconds before puncture and was maintained throughout the cannulation, which generally lasted 10~30 seconds. The tourniquet was removed immediately afterward. Temperature settings were as follows: 40~45°C for the heat therapy group, 0~10°C for the cold therapy group, and alternating heat/cold for the TGI group. In the control group, the tourniquet was used without thermal activation.

Immediately post-venipuncture, Assistant 2—blinded to group allocation—assessed observed pain using the Wong-Baker FACES Pain Rating Scale, measured SpO_2_ on the contralateral arm using a fingertip pulse oximeter, and evaluated the stress index using the Canopy 9 RSA. Participants then completed a post-test questionnaire on perceived pain, stress (NRS), and satisfaction.

### 5. Data Collection and analysis

The period of data collection in this study was April 9–30, 2022, at Daejeon Eulji Medical Center, South Korea. The collected data were statistically analyzed using SPSS version 23.0 (IBM Corp., Armonk, NY, USA). The participants' general characteristics were analyzed using the frequency, real number, percentage, mean, and standard deviation, while the Chi-squared (χ^2^) test and analysis of variance (ANOVA) were performed to test homogeneity. To test the homogeneity of pre-test dependent variables across the four groups (heat, cold, TGI, and control), ANOVA was performed. The reliability of the instrument on TEE tourniquet satisfaction was tested using Cronbach’s α. After the experimental treatments of the four groups, ANOVA was performed to test the levels of perceived pain, stress, and satisfaction. The Bonferroni post-hoc test was performed to test the level of significance.

### 6. Ethical considerations

The study was approved by the Institutional Review Board (IRB) of Daejeon Eulji Medical Center and was approved for protocol modification (IRB No. EMC 2021-01-001). The study was registered with the Clinical Research Information Service (KCT0009336).

Before data collection, recruitment advertisements were posted on the hospital bulletin board with institutional and nursing department approval. Before the intervention, the participants were explained the study’s purpose and procedures, after which signed consent was obtained. The participants were informed that they could drop out of the study at any time without any disadvantages. As the intervention applied the tourniquet that came into contact with the skin, additional explanations on potential side effects (contact dermatitis and others) and their treatments and compensations were provided. In addition, at the end of the intervention, a gift of appreciation was given, which was a voucher worth 10,000 KRW, to the participants in all four groups.

The collected data were coded with an ID number according to guidelines on private information management to ensure privacy protection. The data were used solely for the purpose of the study and stored in a locked box to ensure data security. The data will be discarded after 3 years of storage.

## RESULTS

### 1. General characteristics of the participants and dependent variables and their pre-test homogeneity

A total of 118 participants (29 each in the heat and cold therapy groups and 30 each in the TGI therapy and control groups) were included in this study. The general characteristics and homogeneity test results of the four groups are presented in Table 1. There were 9 men (31.0%) and 20 women (69.0%) in the heat therapy group, 15 men (51.7%) and 14 women (48.3%) in the cold therapy group, 8 men (26.7%) and 22 women (73.3%) in the TGI therapy group, and 13 men (43.3%) and 17 women (56.7%) in the control group, with no significant between-group variation; the mean ages of the participants were 51.83 ± 14.62 years, 45.48 ± 13.94 years, 49.07 ± 12.67 years, and 48.37 ± 12.52 years, respectively.

The most common medical history in the entire study group was surgical disease (n = 60, 50.8%), followed by cardiovascular (n = 38, 32.2%), gynecological (n = 17, 14.4%), endocrine (n = 15,12.7%), pulmonary (n = 13, 11.1%), and gastrointestinal (n = 12, 10.2%) diseases. Similarly, in each group, surgical complication was the most frequent condition in the heat therapy (n = 12, 10.2%), cold therapy (n = 15, 12.7%), TGI therapy (n = 17, 14.4%), and control groups (n = 16, 13.6%), followed by cardiovascular disease in the heat therapy (n = 12, 10.2%), cold therapy (n = 7, 5.9%), TGI therapy (n = 7, 5.9%), and control groups (n = 12, 10.2%). To test the homogeneity of the general characteristics across the four groups, χ^2^-test and ANOVA were performed, and homogeneity was confirmed based on the lack of significant variations in sex, age, height, weight, and current disease.

The results of the homogeneity test for the dependent variables are presented in Table 1. The mean score of perceived pain during venipuncture within the past 6 months was 6.48 ± 1.79 in the heat therapy group, 6.76 ± 1.33 in the cold therapy group, 6.03 ± 2.03 in the TGI therapy group, and 6.23 ± 1.45 in the control group; the mean scores of perceived stress during venipuncture were 6.07 ± 1.85, 5.97 ± 2.38, 5.63 ± 2.80, and 5.13 ± 1.61, respectively. One-way ANOVA was performed to test the homogeneity of the dependent variables across the four groups, and homogeneity was confirmed based on the lack of significant variations in perceived pain and stress levels.

### 2. Effects of TEE tourniquet in venipuncture on pain, SpO_2_, stress, and satisfaction

The effects of the TEE tourniquet on venipuncture pain are shown in Table 2. With the application of the TEE tourniquet, the mean perceived pain scores were 3.31 ± 1.65 in the heat therapy group, 4.24 ± 1.33 in the cold therapy group, 4.10 ± 1.92 in the TGI therapy group, and 5.00 ± 1.88 in the control group. The pain scores displayed significant variation across the four groups (F = 4.82, *p* = .003), with the heat therapy group showing significantly lower scores than the control group. The mean pain scores reported by the observer during venipuncture were 2.69 ± 1.11 in the heat therapy group, 2.97 ± 1.15 in the cold therapy group, 2.73 ± 0.98 in the TGI therapy group, and 3.73 ± 1.26 in the control group, whereby the four groups showed significant variation (F = 5.50, *p* = .001). The post-hoc test indicated that pain scores were significantly lower in the heat therapy and TGI therapy groups than in the control group.

The mean SpO_2_ values measured during the TEE tourniquet venipuncture were 97.72 ± 1.00% in the heat therapy group, 97.72 ± 0.80% in the cold therapy group, 97.53 ± 1.11% in the TGI therapy group, and 97.83 ± 1.18% in the control group; no significant variation was observed (F = 0.44, *p* = .726). The mean satisfaction scores were 39.45 ± 5.81 in the heat therapy group, 38.31 ± 5.39 in the cold therapy group, 38.77 ± 7.27 in the TGI therapy group, and 35.30 ± 6.48 in the control group. Satisfaction was highest in the heat therapy group and lowest in the control group, although the difference was not significant (F = 2.52, *p* = .061).

With the application of the TEE tourniquet, the mean perceived stress scores were 2.45 ± 1.74 in the heat therapy group, 2.86 ± 1.55 in the cold therapy group, 3.50 ± 2.06 in the TGI therapy group, and 4.10 ± 1.84 in the control group. The stress scores displayed a significant variation across the four groups (F = 4.72, *p* = .004), with the heat therapy group showing significantly lower scores than the control group; the mean stress indices measured during venipuncture were 3.21 ± 1.76, 4.31 ± 2.70, 3.87 ± 2.87, and 3.83 ± 2.76, respectively, whereby the four groups showed no significant variation (F = 0.91, *p* = .440).

## DISCUSSION

This study examined the effects of applying heat, cold, and TGI therapies via a thermoelectric tourniquet on pain and stress during venipuncture. While the intervention method was innovative, the primary aim was to explore how these temperature-based modalities influence physiological and subjective responses in clinical procedures.

Pain levels may vary significantly across individuals depending on their tolerance. To ensure comparability, baseline perceived pain levels related to previous venipuncture experiences were assessed, and no significant differences were found across groups, confirming homogeneity.

The TEE tourniquet was applied with heat, cold, and TGI therapy in the respective therapy groups, while it was applied without any temperature therapy in the control group. The results indicated a significant variation in pain across the four groups after the interventions; in particular, the heat therapy group showed a significantly lower level of pain than the control group. The scores of venipuncture pain after the intervention ranged from 3.31 to 5.00, indicating a decrease in all four groups. Notably, the perceived pain in the heat therapy group was reduced by almost 50% compared to the pre-test data, and this difference was significant. The observed pain level was also significantly lower in the heat therapy group than in the control group.

Most previous studies applying heat therapy to reduce venipuncture pain have focused on pediatric patients or those receiving hemodialysis. In this study, the heat therapy group showed significantly lower pain levels than the control group, consistent with earlier findings demonstrating the effectiveness of thermotherapy in reducing pain during needle puncture procedures such as arteriovenous fistula cannulation or abdominal injection [17,34]. Thermotherapy is believed to produce analgesic and sedative effects by interfering with pain transmission. Local heat application increases blood flow, facilitates the removal of metabolic byproducts, and promotes psychological relaxation, all of which contribute to pain reduction (Foulkes & Wood [35]). The results of this study also align with prior studies that used non-pharmacological interventions such as lidocaine spray and aroma massage. This suggests that heat therapy using the TEE tourniquet may provide pain relief comparable to other established methods. Notably, the TEE device offers a practical advantage in clinical settings by enabling rapid and localized heat application, unlike traditional electric heating pads or mats that require longer preparation and application times. On the other hand, although cold therapy led to an average pain reduction of 2.52 points in this study, the difference was not statistically significant compared to the control group. This contrasts with previous research in which cold therapy—such as applying an ice pack for 15 minutes, using vapocoolant spray, or combining cold and vibration—effectively reduced venipuncture pain [12,17,20]. In the current study, the cold stimulus was applied for a shorter period, and only mild cooling (0~10°C) was delivered through the TEE device. Compared to cold sprays that can lower skin surface temperature rapidly to −20°C [36], this may have been insufficient to induce vasoconstriction or slow nerve conduction effectively. According to the gate control theory, inadequate sensory stimulation may fail to activate the large-diameter nerve fibers necessary to "close the gate" at the spinal cord, thereby limiting the analgesic effect. TGI therapy also showed only a limited effect on pain reduction, suggesting that the intensity and duration of stimulation may be critical for achieving analgesic effects.

A previous study using a thermoelectric device reported that cold therapy significantly reduced venipuncture pain [27]. In contrast, this study showed a greater pain-relief effect with heat therapy. Our results agreed with the pain scores observed by the nurse during venipuncture, indicating the occurrence of reduced pain with heat therapy in the temperature range of 40~45°C. The SpO_2_ values in this study did not vary significantly across the four groups, consistent with the results of a previous study that applied the TEE band in venipuncture [27]. Although SpO_2_ was included as a physiological indicator, no significant differences were observed across groups. This may be because the interventions in this study, while effective in reducing subjective pain and stress, were not intensive enough to induce measurable changes in oxygen saturation. On the other hand, cold stimulation has been known to induce peripheral vasoconstriction [37]. However, in this study, no significant differences were found in SpO_2_. Given that SpO_2_ is also related to peripheral perfusion, this result may suggest that cold therapy and TGI therapy were not strong enough to cause vascular changes that could potentially affect intravenous access.

In this study, the pre-intervention stress score based on participants’ venipuncture experiences within the past 6 months was 5.7 on average, and homogeneity was confirmed across the groups. After the intervention, only the heat therapy group showed a significant reduction in subjective stress scores, while no significant difference was observed in objective stress indicators across the four groups. Neither cold nor TGI therapy demonstrated significant effects on either pain or stress. In previous studies, however, cold and TGI therapy were reported to be effective in reducing pain and stress [20,25,31]. These findings suggest that the interventions in this study may not have been strong enough to elicit measurable physiological stress responses. The fact that only heat therapy was effective in reducing both pain and subjective stress may be related to cultural preferences in South Korea, where warmth is traditionally associated with comfort and relief.

The TEE tourniquet developed in this study with the temperature control function allows the stimulation to be felt by the user within approximately 5 seconds of switching on and the target temperature to be reached within only 10 seconds, so it can serve as an effective method of nursing intervention that can be more easily applied than the conventional heat or cold therapy methods. However, the TEE tourniquet with an attached temperature controller was applied for the first time to the patients during venipuncture, and it could have been more effective if the entire arm could have been wrapped with the device by fabricating a larger warming/cooling plate than that in the initial design.

The limitations of this study are as follows. The TEE tourniquet developed in this study combined a flexible thermoelectric device and a tourniquet. This device was applied for the first time in this study, and it allowed the generation of heat and cold depending on the requirement. However, as the device was a prototype, the size was relatively large, and the power depended on the wiring, causing a potential inconvenience to the nurses. Given visual stimulation is an important part of patient treatment, the prototype did not have an aesthetic design. In the experimental design, there was no control group to which the conventional tourniquet was applied. Thus, there could have been a confounder, as the TEE tourniquet control group without temperature stimulation could have had an expected effect solely due to the TEE tourniquet effect. In addition, generalization may be difficult, as the participants were adult hospitalized patients, but this led to increased internal consistency of the study. This could also have been another confounder, considering the diversity of adult hospitalized patients in general wards. Thus, a follow-up study should address the diversities in age, sex, skin sensitivity to temperature, and underlying disease.

Based on the results of this study, the following suggestions are made for future studies applying TEE tourniquets in venipuncture. To maximize the effects of heat or cold therapy when applying the TEE tourniquet, the size of the plate in the flexible thermoelectric device of the TEE tourniquet should be increased to allow the effects to be generated on a larger area of the participant’s skin. To ensure simplified use of the TEE tourniquet, the device should be wireless, and the satisfaction of the individual performing the venipuncture should be investigated. As the level of pain and temperature preference varies across individuals, a tailored intervention is required to address the participant’s characteristics and preferences. In a follow-up study, the effects should be verified through repeated tests at set intervals with identical participants with the same disease or by varying the applied temperature and time.

The TEE tourniquet device in this study had a temperature control function using a flexible thermoelectric device, aimed toward pain relief in venipuncture, a procedure frequently performed at hospitals. The clinical application of the device with heat therapy led to pain and stress relief effects, whereas the applied TEE tourniquet was advantageous in allowing an easier way to apply heat therapy than the conventional electric heating mat. Because the device allows the tourniquet to function, its use is recommended in clinical practice.

The significance of this study lies in applying a thermoelectric tourniquet capable of both heat and cold therapy as a single device during venipuncture. By enabling rapid, localized temperature modulation through electrical energy rather than traditional methods such as hot packs or ice, the device offers a practical and efficient nursing intervention for pain relief.

## CONCLUSION

This study demonstrated that the application of heat therapy using a TEE tourniquet significantly reduced both perceived and observed venipuncture pain, as well as stress levels, in adult patients. The findings support the use of temperature-controlled devices as effective, non-pharmacological interventions for pain and stress management during intravenous catheter insertion. While heat therapy showed clear benefits, cold and TGI therapies yielded limited or inconclusive effects, suggesting the need for further optimization of temperature intensity and application duration.

Clinically, the TEE tourniquet presents a practical advantage over conventional methods due to its rapid thermal response and ease of use, potentially enhancing patient comfort and procedural efficiency. However, as this was a prototype device, improvements in design, portability, and usability are recommended to support broader adoption.

Future studies should explore tailored interventions considering individual preferences and physiological responses and include repeated measures or varied temperature protocols to identify optimal therapeutic parameters. With refinement, the TEE tourniquet has the potential to become a valuable tool in clinical nursing practice for pain relief during venipuncture.

## Figures and Tables

**Table 1.** Homogeneity Test of General Characteristics among the Four Groups (N = 118)

Characteristics	Categories	TEE T-H (n = 29)	TEE T-C (n = 29)	TEE T-TGI (n = 30)	Control (n = 30)	X^2^ or F	*p*
Sex	Men	9 (31.0)	15 (51.7)	8 (26.7)	13 (43.3)	4.91	.179
	Women	20 (69.0)	14 (48.3)	22 (73.3)	17 (56.7)		
Age (yr)		51.83 ± 14.62	45.48 ± 13.94	49.07 ± 12.67	48.37 ± 12.52	1.09	.357
Height (cm)		162.10 ± 8.20	165.86 ± 9.95	162.16 ± 6.48	163.74 ± 9.36	1.24	.299
Weight (kg)		60.45 ± 13.58	67.17 ± 12.51	62.62 ± 12.65	65.18 ± 9.17	1.96	.125
Medical History^†^	Cardio	12 (10.2)	7 (5.9)	7 (5.9)	12 (10.2)	0.87	.462
	Endo	3 (2.5)	5 (4.2)	2 (1.7)	5 (4.2)		
	Pulmo	4 (3.4)	3 (2.5)	3 (2.5)	3 (2.5)		
	Gastro	2 (1.7)	5 (4.2)	3 (2.5)	2 (1.7)		
	Surgical	12 (10.2)	15 (12.7)	17 (14.4)	16 (13.6)		
	Gyneco	7 (5.9)	4 (3.4)	5 (4.2)	1 (0.8)		
Pain (NRS)		6.48 ± 1.79	6.76 ± 1.33	6.03 ± 2.03	6.23 ± 1.45	1.04	.379
Stress (NRS)		6.07 ± 1.85	5.97 ± 2.38	5.63 ± 2.80	5.13 ± 1.61	1.07	.363

Values are presented as the mean ± standard deviation or n (%). TEE T-H = Thermoelectric element tourniquet heat group; TEE T-C = Thermoelectric element tourniquet cold group; TEE T-TGI = Thermoelectric element tourniquet thermal grill illusion group; Control = Control group; Cardio = Cardiovascular disease; Endo = Endocrine system disease; Pulmo = Pulmonary disease; Gastro = Gastrointestinal disease; Surgical = Surgical disease; Gyneco = Gynecological disease; NRS = Numeral rating scale.

† Multiple responses were allowed for medical history.

**Table 2.** Comparison of Dependent Variables among the Four Groups (N = 118)

Variables	TEE T-H (n = 29)	TEE T-C (n = 29)	TEE T-TGI (n = 30)	Control (n = 30)	F	*p*	Bonferroni^†^
Pain (NRS)	3.31 ± 1.65^a^	4.24 ± 1.33^b^	4.10 ± 1.92^c^	5.00 ± 1.88^d^	4.82	.003	a < d*
Obj. Pain (FACES)	2.69 ± 1.11^a^	2.97 ± 1.15^b^	2.73 ± 0.98^c^	3.73 ± 1.26^d^	5.50	.001	a, c < d^*^
Stress (NRS)	2.45 ± 1.74^a^	2.86 ± 1.55^b^	3.50 ± 2.06^c^	4.10 ± 1.84^d^	4.72	.004	a < d^*^
Stress index	3.21 ± 1.76	4.31 ± 2.70	3.87 ± 2.87	3.83 ± 2.76	0.91	.440	
SpO_2_ (%)	97.72 ± 1.00	97.72 ± 0.80	97.53 ± 1.11	97.83 ± 1.18	0.44	.726	
Satisfaction	39.45 ± 5.81	38.31 ± 5.39	38.77 ± 7.27	35.30 ± 6.48	2.52	.061	

Values are presented as the mean ± standard deviation. TEE T-H = Thermoelectric element tourniquet heat group; TEE T-C = Thermoelectric element tourniquet cold group; TEE T-TGI = Thermoelectric element tourniquet thermal grill illusion group; Control = Control group; NRS = Numeral rating scale; FACES = Wong-Baker FACES Pain Rating Scale; SpO_2_ = Saturation of percutaneous oxygen.

^†^Bonferroni-adjusted significant results are presented; ^*^p ≤ .05, Bonferroni.

## Data Availability

The data that support the findings of this study are available from the corresponding author upon reasonable request.
